# Targeting Gastric Cancer Stem Cells to Enhance Treatment Response

**DOI:** 10.3390/cells11182828

**Published:** 2022-09-10

**Authors:** Xionghui Rao, Chaojun Zhang, Huixing Luo, Jianbao Zhang, Zhehong Zhuang, Zhihao Liang, Xiaobin Wu

**Affiliations:** Department of Gastrointestinal Surgery, The Eighth Affiliated Hospital of Sun Yat-Sen University, Shenzhen 528406, China

**Keywords:** gastric cancer stem cells, treatment

## Abstract

Gastric cancer (GC) was the fourth deadliest cancer in the world in 2020, and about 770,000 people died from GC that year. The death of patients with GC is mainly caused by the metastasis, recurrence, and chemotherapy resistance of GC cells. The cancer stem cell theory defines cancer stem cells (CSCs) as a key factor in the metastasis, recurrence, and chemotherapy resistance of cancer. It considers targeting gastric cancer stem cells (GCSCs) to be an effective method for the treatment of GC. For GCSCs, genes or noncoding RNAs are important regulatory factors. Many experimental studies have found that some drugs can target the stemness of gastric cancer by regulating these genes or noncoding RNAs, which may bring new directions for the clinical treatment of gastric cancer. Therefore, this review mainly discusses related genes or noncoding RNAs in GCSCs and drugs that target its stemness, thereby providing some information for the treatment of GC.

## 1. Introduction

GC is a common malignant tumor. In 2020, cancer caused 10 million deaths in the world, and GC accounted for 7.7% (770,000), ranking the fourth cause of death after lung cancer, colorectal cancer, and liver cancer [1]. Although there are various treatments for GC, the survival rate of many advanced GC patients with metastasis, recurrence, and chemotherapy is quite low [2,3,4,5,6,7,8,9]. Therefore, new therapies are urgently required to improve the survival rate of advanced GC patients.

**Figure 1:**
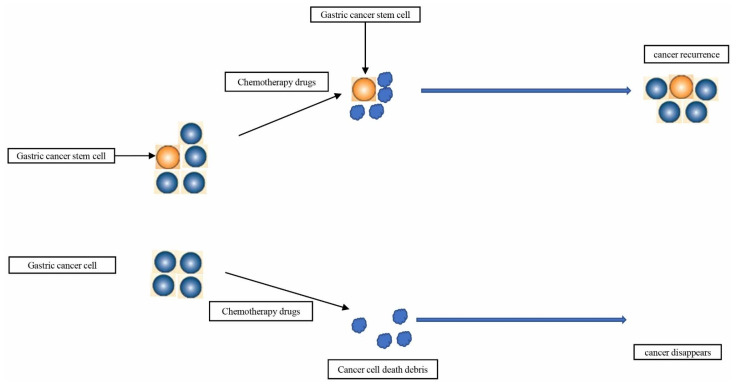
GCSCs are also considered to be a key factor in GC recurrence and chemotherapy resistance.

Many researchers find that cancer stem cells may be a key factor in the treatment of advanced cancer. For instance, T Lapidot et al. found that a large number of cell colonies that they called leukemia-initiating cells could be generated by transplanting a small number of CD34+CD38- leukemia cells into mice [10]. T Reya et al. believed that CSCs are the key to cancer treatment. If the CSCs are not killed, but only ordinary cancer cells are killed, the cancer will soon relapse [11]. American Association for Cancer Research (AACR) in 2006 defined that CSCs in tumors have the ability to self-renew and generate heterogeneous tumor cells. Experts at the meeting believed that this cell subset may be resistant to classical treatments, and new drugs need to be developed to selectively target cancer stem cells to treat cancer [12]. With the gradual deepening of research on CSCs, it has been discovered that CSCs are the initiating cells of malignant tumors. They play a key role in cancer metastasis, recurrence, and chemotherapy resistance [13,14,15,16]. In view of the important role of CSCs in GC, many scholars believe that the key to the treatment of GC is to completely eliminate CSCs in gastric cancer [17,18,19] (Figure 1). In order to improve the prognosis of patients with advanced GC, finding new drugs to target GCSCs may be the direction of therapy [20,21]. Genes can regulate the stemness of GC cells, and some drugs target the stemness of GC cells by changing the expression of these gastric, cancer, stem cell-related genes [22,23,24,25,26]. Therefore, in this review, we focused on related genes in GCSCs and drugs and provide some information to treat GC by targeting its stemness.

## 2. The Origin, Isolation, Surface Markers, and Related Signaling Pathways of GCSCs

The origin of GCSCs is currently somewhat controversial. Many researchers held the view that GCSCs might be derived from gastric stem cells. S M Karam et al. found that TFF1 knockout mouse gastric stem cells contribute to gastric carcinogenesis [27]. Lang Yang et al. proved that GC stem-like cells possess higher capability of invasion and metastasis in association with a mesenchymal transition phenotype [28]. These evidences indicated that GCSCs may be derived from gastric stem cells. However, bone marrow-derived cells (BMDCs) discovered by Shigeo Takaishi et al. during their research using a mouse model of Helicobacter-induced GC may also be a source of GCSCs. Meanwhile, BMDCs are considered to be the most primitive uncommitted adult stem cells [29]. Therefore, the source of GCSCs may be gastric stem cells or BMDCs.

At present, there are three mainstream methods for the isolation of GCSCs. The first method is that GCSCs can be separated from side population (SP) cells using nuclear fluorescent dyes. GCSCs have the ability to expel Hoechst33342 so they cannot be stained. The main group of cells is the smaller cell group at the lower left; they are often located in the dot plot of flow analysis. According to this characteristic, the GCSCs can be obtained and collected by selecting the appropriate wavelength of ultraviolet excitation light by the flow cytometer with a sorting function [20]. The second method is to use the surface markers of GCSCs for cell sorting. By applying magnetic strain-activated cell sorting (MACS) technology, Shigeo Takaishi et al. used CD44+ as the surface marker of GCSCs to separate them. The specific mechanism is that the CD44 antibody is fixed on the separation column, and the CD44 positive cells can be adsorbed on the CD44 antibody on the separation column to avoid being repelled from the separation system by the magnetic field [30]. Of course, there are also other surface markers GCSCs that will be discussed later. The third method is to isolate GCSCs by serum-free low-adherence culture. When being cultured in a serum-free, low-adherence medium supplemented with growth factors, GCSCs can form spheroid cells and maintain self-renewal properties, while normal GC cells cannot survive. Spheroid formation assay is considered as a convenient way to obtain GC [20].

It is currently believed that the common surface markers of GCSCs include CD44, CD133, LGR5, MIST1, ALDH1, and AQP5. CD44, CD133, and ALDH1 are also markers for many other cancer stem cells. LGR5 is a G protein-coupled receptor with a seven-pass transmembrane structure. It is considered to be enriched in gastric cancer stem cells. Gastric stem cells expressing Mist1 are thought to have a propensity to transform into gastric cancer stem cells. AQP5 is thought to be enriched in distal gastric cancer stem cells [18,31,32,33,34]. GCSCs can be isolated by fluorescence-activated cell sorting (FACS) or MACS using the above surface markers.

There are many signaling pathways involved in GCSCs. The most common ones include WNT signaling pathway, NOTCH signaling pathway, Hedgehog signaling pathway, and HIPPO signaling pathway [35,36,37,38]. It is well known that the most common signaling pathways of CSCs are WNT, NOTCH, and Hedgehog [39,40,41,42,43]. Aberrant Wnt/β-catenin signaling promotes CSCs renewal, cell proliferation, and differentiation, so they play a critical role in tumorigenesis and therapeutic response [44]. In various cancer types, NOTCH signaling triggers CSCs phenotypes that develop resistance to various therapies, thus potentially leading to cell dormancy and relapse [45]. Hedgehog signaling, a developmental pathway that is mostly inactive in adult tissues except for stem cells, is frequently found to be upregulated in various tumors and is associated with CSCs maintenance [46]. However, in GCSCs, HIPPO signaling pathway is also a very common signaling pathway [47].

## 3. The Role of Protein-Encoding Genes in GCSCs

Targeted therapy improves the prognosis of cancer patients and brings hope to them [48]. For example, trastuzumab, which targets the *HER2* gene, improves the survival rate of patients with HER2-positive breast cancer [49]. Gefitinib could target EGFR to improve the survival rate of patients with small cell lung cancer [50], and VEGFR-targeting bevacizumab improves the survival rate of colorectal cancer patients [51]. Although there are currently no marketed drugs targeting CSCs, researchers have found that targeting CSCs is a feasible way to treat tumors [15]. Rui Su et al. discovered that small molecules CS1 and CS2 targeting FTO can inhibit the renewal of tumor stem cells [24]; Cheng Wang et al. found that anti-CD276 antibody inhibits squamous cell carcinoma stem cells [52]; Yufeng Shi et al. found that gboxin is an oxidative phosphorylation inhibitor that targets glioblastoma [53]. The above results showed that targeting genes with inhibitors or antibodies to inhibit CSCs has potential in treating tumors, which is also applicable in GC. Chien-Hsing Lee et al. found that liquiritigenin inhibited the stem cell-like characteristics of GC by down-regulating the expression of glucose-regulated protein 78 and inhibiting the growth of GC [54]. Therefore, inhibiting GC stemness by inhibiting the expression of GCSCs-related genes is also a method for treating GC.

The protein-encoding gene plays a very important role in the regulation of GCSCs. From the introduction, the common signaling pathways of GCSCs include WNT, NOTCH, Hedgehog, and HIPPO signaling pathways. These genes that regulate GCSCs were divided into two parts: through these above common signaling pathways and not through the above common signaling pathway.

### 3.1. Related Protein-Encoding Genes in GCSCs through WNT, NOTCH, Hedgehog, and HIPPO Signaling Pathway

**Figure 2:**
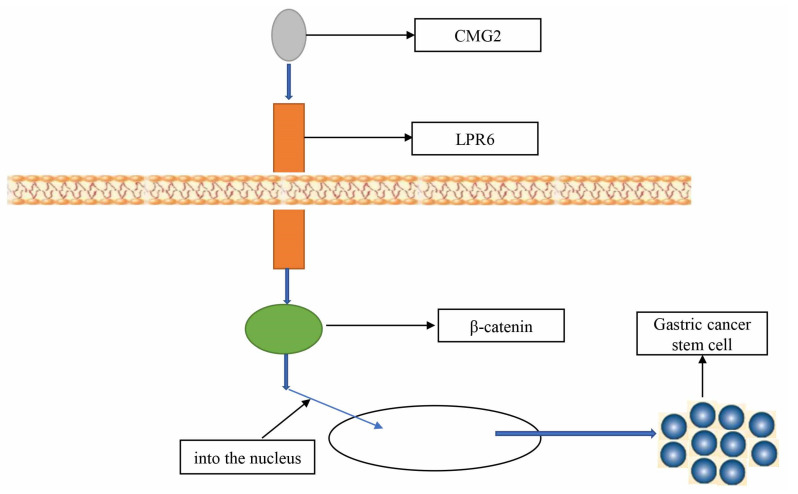
CMG2 maintains GC stem-like cell phenotype by activating a Wnt/β-catenin pathway.

Many genes can regulate the stemness of GC cells by affecting WNT, NOTCH, Hedgehog, and HIPPO signaling pathways (Table 1). The WNT signaling pathway is a very important pathway in GCSCs. Based on Kaiyun Guo et al., tumor necrosis factor-α-inducible protein (Tipα) promotes the tumor stem cell-like properties of GC cells by activating the Wnt/β-catenin signaling pathway, thereby accelerating the progression of GC, and targeting Tipα may be the strategy to treat GC [55]. According to Chengdong Ji et al., capillary morphogenesis gene 2 (*CMG2*) is highly expressed in GC, and CMG2 interacts with LRP6 in GCSCs to activate the Wnt/β-catenin pathway. Their results revealed that CMG2 promotes GC by maintaining GCSCs progression and may serve as a new prognostic marker and target for human GC therapy [56] (Figure 2). Yunhe Gao et al. found that the expression of ring finger protein 43 (RNF43) was decreased in GCSCs. RNF43 could inhibit the stemness of GC cells by inhibiting the Wnt/β-catenin pathway, the specific mechanism is that RNF43 upregulates the expression of Lgr5 protein, an upstream activator of WNT signaling pathway [57].

NOTCH signaling pathway also plays an important role in GCSCs. Yan Dou et al. found that the expression of NOTCH1 was increased in GC, the overexpression of NOTCH1 increases the stemness of GC cells, and the knockdown of NOTCH1 reduces the stemness of GC cells. They believed that targeting inhibition of NOTCH signaling pathway on the human GCSCs has drug resistance [58]. Zhi-Feng Miao et al. proposed that the expression of DLL4 was increased in 383 GC tissue samples and was associated with the risk of distant metastasis. DLL4 silencing inhibited the self-renewal of GCSCs and enhanced the multi-differentiation ability, which was achieved through the NOTCH signaling pathway [59].

Hedgehog and HIPPO signaling pathway also affects GCSCs. Yixun Lu et al. found that GLI2 is highly expressed in GC and promotes the stemness of GC cells through the Hedgehog signaling pathway [60]. According to Yunhe Gao et al., the expression of stearoyl-CoA desaturase 1 (SCD1) was increased in metastatic GC, and SCD1 promoted the stem cell-like properties of GCSCs, which was achieved by affecting the expression of YAP and promoting the HIPPO pathway. Therefore, they believe that targeting SCD1 may be a new therapeutic strategy, especially to inhibit GC metastasis and improve chemosensitivity [61].

Many other genes affect GCSCs through WNT, NOTCH, Hedgehog, and HIPPO signaling pathways, which we have summarized in Table 1 [62,63,64,65,66,67,68,69].

**Table 1:** Related protein-encoding genes in GCSCs through WNT, NOTCH, Hedgehog, and HIPPO signaling pathways.

Genes	Functions	Mechanism	Reference
*Tipα*	Promote stemness	Wnt/β-catenin signaling pathway	[55]
*CMG2*	Promote stemness	Wnt/β-catenin signaling pathway	[56]
*RNF43*	Inhibit stemness	Wnt/β-catenin signaling pathway	[57]
*NOTCH1*	Promote stemness	NOTCH signaling pathway	[58]
*DLL4*	Promote stemness	NOTCH signaling pathway	[59]
*GLI2*	Promote stemness	Hedgehog signaling pathway	[60]
*SCD1*	Promote stemness	HIPPO signaling pathway	[61]
*RORβ*	Inhibit stemness	Wnt/β-catenin signaling pathway	[62]
*PIGF*	Promote stemness	Wnt/β-catenin signaling pathway	[63]
*NANOGP8*	Promote stemness	Wnt/β-catenin signaling pathway	[64]
*HES1*	Promote stemness	NOTCH signaling pathway	[65]
*PAR1*	Promote stemness	HIPPO signaling pathway	[66]
*WNT1*	Promote stemness	Wnt/β-catenin signaling pathway	[67]
*Dickkopf-1*	Inhibit stemness	Wnt/β-catenin signaling pathway	[68]
*TAK1*	Promote stemness	HIPPO signaling pathway	[69]

### 3.2. Genes Related to GCSCs-Encoded Proteins with Unspecified Mechanisms or Not Acting through WNT, NOTCH, Hedgehog, and HIPPO Signaling Pathways

Researchers also found many genes related to GCSCs-encoded proteins with unspecified mechanisms or not acting through WNT, NOTCH, Hedgehog, and HIPPO signaling pathways. Some related gene researchers have not explained the mechanism through metabolism, other signaling pathways, hypoxia induction, autophagy, etc.

On the basis of Li-Fei Sun et al., *HER2* knockdown in GCSCs reduced the self-renewal, proliferation, colony formation, chemoresistance, and invasion and migration abilities of GCSCs [70]. Natalia Pajuelo-Lozano et al. found that *MAD2* is important for the stemness of GCSCs, and its downregulation in GCSCs plays a central role in the occurrence of GC [71]. In addition, other researchers have discovered the role of TRAF6, TAZ, α2δ1 subunit, LINGO2, ALDH, B7-H1, RegIV, and CDK5RAP3 in GCSCs. However, the mechanism is currently unknown, and we have summarized them in Table 2 [72,73,74,75,76,77,78,79].

It is currently believed that the metabolism of CSCs is heterogeneous, and the four key metabolisms of CSCs are glucose metabolism, glutamine metabolism, mitochondrial metabolism, and lipid metabolism [80,81,82]. Kai Nie et al. found that GCSCs consume more glutamine than ordinary GC cells. The glutamine transporter *SNAT2* is highly expressed in GCSCs, and SNAT2 overexpression significantly increases the stemness of GC cells [83]. Ting Yang et al. found that Enolase 1 is highly expressed in GCSCs, and Enolase 1 promotes the stemness, metastatic ability, and chemoresistance of GC cells through glycolysis [84].

Many other signaling pathways are also involved in the progression of CSCs, such as ERK, JAK/STAT, and NF-KB signaling pathways [85,86,87]. Jun Lu et al. found that TBL1XR1 promotes the stemness and metastatic ability of GC cells through the ERK signaling pathway [88]. Xiao-Feng Xu et al. discovered that BMX-ARHGAP fusion protein can promote the stemness of GC cells through the JAK/STAT3 signaling pathway. The mechanism is that BMX-ARHGAP activates JAK/STAT signaling by increasing the expression of BMX-SH2 protein, which contains SH2 domain by binding to phosphorylated tyrosine residues [89]. Y-X Jiang et al. also argued that IL-17 promotes GC stemness through STAT3 signaling pathway [90]. Myoung-Eun Han et al. found that NRG1 secreted by CAFs promotes the self-renewal of GCSCs through the NF-KB signaling pathway [91].

Many studies have indicated that hypoxia can induce the enrichment of CSCs and promote the stemness of cancer cells [92]. Shi-Wei Yang et al. found that after hypoxia treatment, compared with normoxic controls, some GCSCs significantly exhibited the increased expression of hypoxia-inducible factor 1α (HIF-1α), increased migration and invasion abilities, and up-regulated HIF-1α that caused GC recurrence and metastasis by activating Snail [93]. Zhi-Feng Miao et al. also found that HIF-1α can promote the stemness of GC cells [94].

Autophagy is the non-selective degradation of cells and the phagocytosis of damaged and denatured proteins, lipids, organelles, and intracellular pathogens in the cytoplasm. Use of degradation could produce energy and raw materials [95]. Autophagy is activated in CSCs, and autophagy promotes the stemness of cancer cells [96]. Shingo Togano et al. agreed that GCSCs survive in stress environments via their autophagy system [97]. Sarah Courtois et al. found that autophagy induced by Helicobacter pylori infection is necessary for GCSCs emergence [98]. Hitoshi Tsugawa et al. found that CagA autophagic degradation is specifically inhibited in cancer stem-like cells [99].

The above discussion does not cover all; we have summarized the remaining genes in Table 2 [100,101,102,103,104,105,106].

**Table 2:** Genes related to GCSCs-encoded proteins with unspecified mechanisms or not acting through WNT, NOTCH, Hedgehog, and HIPPO signaling pathways.

Genes	Functions	Mechanism	Reference
*HER2*	Promote stemness	-	[70]
*MAD2*	Promote stemness	-	[71]
*TRAF6*	Promote stemness	-	[72]
*TAZ*	Promote stemness	-	[73]
*α2δ1*	Promote stemness	-	[74]
*LINGO2*	Promote stemness	-	[75]
*ALDH*	Promote stemness		[76]
*B7-H1*	Promote stemness	-	[77]
*RegIV*	Promote stemness	-	[78]
*CDK5RAP3*	Promote stemness	-	[79]
*SNAT2*	Promote stemness	glutamine	[83]
*Enolase 1*	Promote stemness	glycolysis	[84]
*TBL1XR1*	Promote stemness	ERK signaling pathway	[88]
*BMX-ARHGAP*	Promote stemness	JAK/STAT3 signaling pathway	[89]
*IL-17*	Promote stemness	STAT3 signaling pathway	[90]
*NRG1*	Promote stemness	NF-KB signaling pathway	[91]
*HIF-1α*	Promote stemness	Snail	[93]
*CagA*	Promote stemness	autophagy	[99]
*METTL3*	Promote stemness	PARP1	[100]
*NME2*	Promote stemness	apoptosis	[101]
*KDM4C*	Promote stemness	ALDH1A3	[102]
*E2F1*	Promote stemness	CD44	[103]
*SNAIL*	Promote stemness	CCN3, NEFL	[104]
*SLC34A2*	Promote stemness	miR-25/Gsk3β	[105]
*ATOH1*	Inhibit stemness	differentiation of CSCs	[106]

## 4. The Role of Related Non-Coding RNAs in GCSCs

Noncoding RNAs play important regulatory roles in various diseases, such as cancers [107,108,109,110,111].

Nadya Dimitrova et al. found that miR-143/145 promotes lung cancer progression by targeting CAMK1D [112]. Yina Qiao et al. found that Lnc-408 acts as a sponge for miR-654-5p to alleviate miR-654-5p inhibition of its target LIMK1 and promote breast cancer cell invasion and metastasis [113]. Of course, non-coding RNAs also play regulatory roles in CSCs. For example, Junko Mukohyama et al. found that MIR-221 enhanced the tumorigenicity of human colorectal CSCs by targeting QKI. The regulation of non-coding RNA in GCSCs is mainly miRNA and LncRNA, and there are almost no reports on circular RNA. As such, we will discuss the role of non-coding RNA in GCSCs from miRNA and LncRNA, respectively.

### 4.1. The Role of MiRNA in GCSCs

MiRNAs are RNAs approximately 22 nucleotides in length that silence gene expression post-transcriptionally by binding to the 3′ untranslated region of the target mRNA [114]. The mechanism of the action of miRNA in GCSCs is mostly by inhibiting the expression of GC cell stemness genes.

Chen Shen et al. found that miR-15a-5p was down-regulated in GCSCs, and inhibited the stemness of GC cells by targeting *ONECUT2* [115]; Yixun Lu et al. found that miR-144-3p was down-regulated in GC and combined with the 3′ untranslated region-AUACUGU of 1689–1696 of *GLI2* to inhibit the stemness of GC cells [60]. Panpan Zhan et al. found that miR-98-5p was down-regulated in GCSCs and inhibited the self-renewal, invasion, tumorigenicity, and paclitaxel chemosensitivity of GCSCs by targeting *BCAT1* [116]. Haiwei Ni et al. found that miR-375 mainly targets *SLC7A11* to attenuate the stemness of GC cells [117].

There are currently a variety of miRNA-derived clinical nucleotide drugs (mdCND) in clinical trials, and it is believed that mdCND can benefit patients clinically in the near future [118]. Many other miRNAs also play a role in GCSCs, and we have summarized this in Table 3 [119,120,121,122,123,124,125,126,127,128,129,130,131,132].

**Table 3:** Related miRNAs in GCSCs.

miRNAs	Functions	Mechanism	Reference
miR-15a-5p	Inhibit stemness	*ONECUT2*	[115]
miR-144-3p	Inhibit stemness	*GLI2*	[60]
miR-98-5p	Inhibit stemness	*BCAT1*	[116]
miR-375	Inhibit stemness	*SLC7A11*	[117]
miR-451b	Inhibit stemness	-	[119]
miR-17-5p	Promote stemness	*MKL-1*	[120]
miR-6778-5p	Promote stemness	*YWHAE*	[121]
miR-7-5p	Inhibit stemness	*Smo, Hes1*	[122]
miRNA-598	Inhibit stemness	*RRS1*	[123]
miRNA-193a-3p	Promote stemness	*SRSF2*	[124]
miRNA-19b/20a/92a	Promote stemness	*E2F1, HIPK1*	[125]
miR-132	Promote stemness	*SIRT1*	[126]
miR-196a-5p	Promote stemness	*Smad4*	[127]
miRNA-145	Inhibit stemness	*CD44*	[128]
miR-501-5p	Promote stemness	*DKK1, NKD1, GSK3β*	[129]
miR-483-5p	Promote stemness	-	[130]
miR-106b	Promote stemness	*Smad7*	[131]
miR-34	Inhibit stemness	*Bcl-2*	[132]

### 4.2. The Role of IncRNA in GCSCs

Long non-coding RNA (lncRNA) is a non-coding RNA with a length of more than 200 nucleotides. There are many mechanisms of the action of lncRNA, and the most common mechanism is to act as a miRNA sponge to relieve the inhibitory effect of miRNA on target genes [133,134,135].

Yuanjian Hui et al. found that the expression of LncRNA FEZF1-AS1 was increased in GC tissues and cells. The inhibitory effect of miR-363-3p on *HMGA2* was relieved by adsorbing miR-363-3p, which promoted the progress of GCSCs [136]. Haiyang Zhang et al. found that lncFERO can inhibit the stemness of GC cells by promoting the expression of *SCD1* [137]. Shuai Wang et al. also found that lncRNA ROR promoted the stemness of GC cells [138] (Table 4).

**Table 4:** Related lncRNAs in GCSCs.

lncRNAs	Functions	Mechanism	Reference
LncRNA FEZF1-AS1	Promote stemness	miR-363-3p	[136]
lncFERO	Inhibit stemness	*SCD1*	[137]
lncRNA ROR	Promote stemness	-	[138]

## 5. Current Therapies Targeting CSCs

Although there are currently no FDA-approved drugs for clinical use in CSCs, many drugs targeting CSCs that are in clinical trials have shown promising results (Table 5).

Vantictumab is an antagonist of the WNT signaling pathway, and its specific mechanism is that it can bind to the extracellular segment of the FZD receptor conserved antigen to inhibit WNT signaling induced by multiple WNT family members. Austin Gurney et al. found that vantictumab can reduce tumor-initiating cell frequency [139]. A combination of vantictumab and taxane sensitizes CSCs to taxanes [140]. The combination of vantictumab and paclitaxel demonstrated significant efficacy in a phase Ib clinical study in patients with metastatic breast cancer [141]. Ipafricept is also an anticancer stem cell drug that acts through the WNT signaling pathway [142]. Ipafricept in combination with gemcitabine and paclitaxel demonstrates a high rate of clinical benefit in phase Ib trial in patients with stage IV pancreatic cancer [143].

MK0752 is an inhibitor of NOTCH signaling pathway, and its specific mechanism is to inhibit the activation of NOTCH intracellular segment by inhibiting γ-secretase, thereby inhibiting the expression of NOTCH downstream genes. In a clinical trial of 30 breast cancer patients treated with MK0752 in combination with docetaxel, reductions in CD44(+)/CD24(−), ALDH(+) and mammosphere formation efficiency were observed in their tumors [144].

Hedgehog inhibitor vismodegib was approved by the FDA in 2012 for the treatment of basal cell carcinoma [145]. Edward J Kim et al. found that in a clinical trial of vismodegib combined with gemcitabine in patients with metastatic pancreatic cancer, the expression of GLI1 and PTCH1 was down-regulated, but there was no significant change in pancreatic cancer stem cells [146].

The results of the above clinical trials targeting CSCs are a promising therapeutic approach, which requires more large-scale clinical trials to validate.

**Table 5:** Current therapies targeting CSCs.

Drugs	Functions	Mechanism	Phase of Clinical	Reference
Vantictumab	Inhibit stemness	WNT	Ib	[141]
Ipafricept	Inhibit stemness	WNT	Ib	[143]
MK0752	Inhibit stemness	NOTCH	clinical trial	[144]
vismodegib	Inhibit stemness	Hedgehog	clinical trial	[146]

## 6. Drugs for GC Treatment by Targeting Its Stemness

The emergence of chemotherapy drugs has greatly improved the survival rate of patients with cancer. The aforementioned trastuzumab, gefitinib, and bevacizumab have benefited many patients with cancer [48,49,50,51]. Although there are currently no FDA-approved drugs or drugs entering clinical trials, many basic experiments have shown that drugs or new material can play a role in the treatment of GC by targeting the stemness of GC. We will discuss that separately below.

### 6.1. Drugs for GC Treatment by Targeting Its Stemness

The researchers have discovered many drugs that could target the stemness of GC, including marketed chemotherapy drugs, clinical drugs for other diseases, small molecule drugs, and traditional Chinese medicines (TCM).

Other cancer chemotherapy drugs also play a role in GCSCs. Wanshuang Cao et al. found that Apatinib can inhibit the stemness of GC cells through the Hedgehog signaling pathway. The specific mechanism is that Apatinib acts by inhibiting the key protein SMO in the Hedgehog signaling pathway [147]. P H Nguyen et al. found that all-trans retinoic acid targets GCSCs and inhibits patient-derived gastric carcinoma tumor growth. The mechanism is that all-trans retinoic acid downregulates the expression of CSC markers, CD44 and ALDH, and stemness genes, such as *Klf4* and *Sox2*, and induces tumorsphere differentiation [148]. ERBB2 is overexpressed in approximately 25% of gastric primary tumor models, which correlates with higher levels of CD90 expression in these tumors, and CD90(+) cells have a higher ability to initiate tumors in vivo. J Jiang et al. found that trastuzumab inhibits the stemness of GC cells by inhibiting ERBB2 signaling [149]. The stemness inhibitory effect of cisplatin in GC was discovered by Yang Han et al. [150].

**Figure 3:**
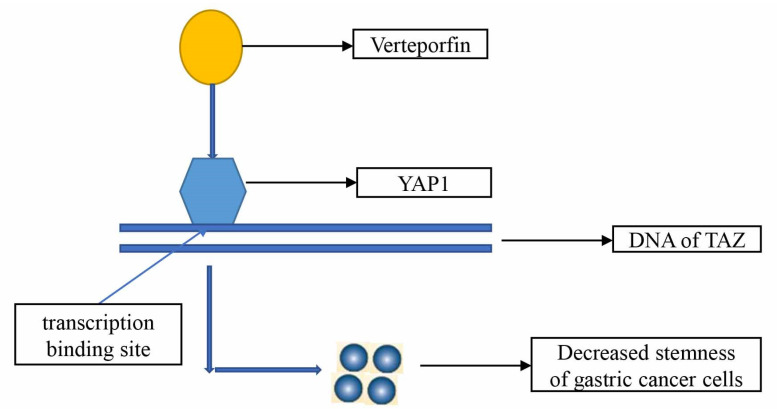
Verteporfin inhibits tumorigenic properties of GCSCs by targeting YAP1/TAZ transcriptional activity.

Drugs clinically used for non-tumor therapy can also inhibit the stemness of GC cells. Atsushi Shiozaki et al. found that amlodipine and verapamil inhibited the growth of GCSCs [151]; Julie Giraud et al. found that Verteporfin inhibited the tumorigenic properties of GCSCs by targeting YAP1/TAZ-TEAD transcriptional activity [152] (Figure 3); Jixian Xiong et al. also found that Verteporfin over-regulated HSP90 function to inhibit the stemness of GC cells [153]; and Hassan Akrami et al. found that ibuprofen can inhibit the stemness of GC cells by inhibiting the Wnt/β-catenin signaling pathway [154]. The researchers also found that metformin and pantoprazole inhibit GC stemness, though the authors did not explain the mechanism by which metformin inhibits the stemness of GC, while the mechanism by which pantoprazole inhibits the stemness of GC is through the EMT/β-catenin pathway [155,156].

Many newly discovered small-molecule drugs also play a role in suppressing the stemness of GC cells. Yun-Shen Tai et al. found that 4′-bromoresveratrol (4-BR) inhibited GC cell stemness through the SIRT3-c-Jun N-terminal kinase pathway [157]; Yao-Dong Zhu et al. found that Celastrus orbiculatus extract (COE) inhibited the stemness of GC cells by regulating the expression of PDCD4 and EIF3H [158]; Xinsheng Shen et al. found that Quercetin triggered mitochondrial apoptosis-dependent growth inhibition by inhibiting PI3K/Akt signaling to play a role in suppressing the stemness of GC cells [159]. Of course, the researchers have also discovered many other small molecule drugs to inhibit the stemness of GC cells, which provided an experimental basis for the treatment of GC [160,161,162,163,164,165,166] (Table 6).

It has also been reported that TCM could inhibit the stemness of GC cells. Yue-Jun Li et al. found that Sijunzi Decoction inhibited the stemness of GC cells by inhibiting the transcriptional activity of β-Catenin [167]. Furthermore, Bing Yan et al. found that Xiaotan Sanjie decoction inhibited GC cell stemness and angiogenesis through Notch1 [168].

Although the drugs described above have not entered clinical trials, they have shown good therapeutic effects targeting GCSCs during in vivo and in vitro trials. It is believed that some of the above drugs would enter clinical trials and be approved by the FDA in the near future and benefit GC patients.

**Table 6:** Drugs for GC treatment by inhibiting its stemness.

Drugs	Functions	Mechanism	Reference
apatinib	Inhibit stemness	Hedgehog signaling pathway	[147]
all-trans retinoic acid	Inhibit stemness	-	[148]
trastuzumab	Inhibit stemness	ERBB2 signaling	[149]
cisplatin	Inhibit stemness	-	[150]
amlodipine verapamil	Inhibit stemness	-	[151]
Verteporfin	Inhibit stemness	YAP1/TAZ	[152]
Verteporfin	Inhibit stemness	HSP90	[153]
ibuprofen	Inhibit stemness	Wnt/β-catenin signaling pathway	[154]
Metformin	Inhibit stemness	-	[155]
pantoprazole	Inhibit stemness	EMT/β-catenin pathways	[156]
4-BR	Inhibit stemness	SIRT3-c-Jun N-terminal kinase pathway	[157]
COE	Inhibit stemness	PDCD4, EIF3H	[158]
Quercetin	Inhibit stemness	PI3K/Akt signaling	[159]
PTPRU	Inhibit stemness	Hippo/YAP Signaling Pathway	[160]
Sulforaphane	Inhibit stemness	hedgehog pathway	[161]
DFOG	Inhibit stemness	FoxM1	[162]
Evodiamine	Inhibit stemness	Wnt/β-catenin signaling pathway	[163]
DAPT	Inhibit stemness	Notch pathway	[164]
Atractylenolide I	Inhibit stemness	Notch pathway	[165]
Genistein	Inhibit stemness	ERK	[166]
Sijunzi Decoction	Inhibit stemness	transcriptional activity of β-Catenin	[167]
Xiaotan Sanjie decoction	Inhibit stemness	Notch1	[168]

### 6.2. New Material for GC Treatment by Targeting Its Stemness

Many new materials have played a huge role in treating cancer. For example, Doxil, the first FDA-approved liposome drug, has shown great success in the treatment of ovarian cancer and breast cancer [169,170,171,172]. Vincristine sulfate liposome injection Marqibo was also FDA-approved for the treatment of adults with advanced, relapsed, and refractory Philadelphia chromosome-negative ALL [173,174]. The emergence of these drugs proved that new materials drugs have great potential in cancer treatment. Therefore, many researchers have pointed out that new materials drugs can inhibit GC cells by targeting the stemness of GC cells, which provides a research basis for the treatment of GC.

Hongjuan Yao et al. found that Gli1 siRNA nanoparticles inhibited the stemness of GC cells by inhibiting the Hedgehog signaling pathway, which provided a promising targeted therapy strategy for the treatment of GC [175]; Han Chen et al. found that nanoparticles CD44/CD133-ATRA-PLPN can inhibit the proliferation of GCSCs [176]; Feng Yang et al. found that CD44 targeting USP22 small interfering RNA-loaded nanoliposomes can target and eradicate GCSCs [177]; Weifeng Yang et al. found that HA-coated nanoparticles, co-encapsulating plasmid METase, and 5-Fu showed enhanced application in targeting GCSCs [178]. Many other new material drugs could inhibit the stemness of GC cells [179,180,181]. These new materials have also shown very good therapeutic effects in experiments targeting GCSCs (Table 7), and it is expected that they will benefit cancer patients like Doxil.

**Table 7:** New materials for GC treatment by inhibiting its stemness.

New Materials Drugs	Functions	Mechanism	Reference
Gli1 siRNA nanoparticles	Inhibit stemness	Hedgehog signaling pathway	[175]
CD44/CD133-ATRA-PLPN	Inhibit stemness	-	[176]
USP22-NLs-CD44	Inhibit stemness	-	[177]
METase/5-Fu co-encaspulated NPs	Inhibit stemness	-	[178]
miR-34a delivery system	Inhibit stemness	CD44	[179]
CD44v6-GNS nanoprobes	Inhibit stemness	-	[180]
SAL-SWNT-CHI-HA complexes	Inhibit stemness	-	[181]

## 7. Conclusions

GC is a disease that plagues the world. The metastasis, recurrence, and chemotherapy resistance of GC are very fatal to GC patients. According to the GCSCs theory, GCSCs play a key role in the metastasis, recurrence, and chemotherapy resistance of GC, and GC stemness genes can regulate GCSCs. It may serve as a way to treat GC by targeting the expression of GC stemness genes. Therefore, we have summarized the related genes or noncoding RNAs in GCSCs and drugs for GC treatment by targeting its stemness, which could provide some information for the clinical treatment of GC.
